# Quantitative evaluation of the influence of multiple MRI sequences and of pathological tissues on the registration of longitudinal data acquired during brain tumor treatment

**DOI:** 10.3389/fnimg.2022.977491

**Published:** 2022-09-20

**Authors:** Luca Canalini, Jan Klein, Diana Waldmannstetter, Florian Kofler, Stefano Cerri, Alessa Hering, Stefan Heldmann, Sarah Schlaeger, Bjoern H. Menze, Benedikt Wiestler, Jan Kirschke, Horst K. Hahn

**Affiliations:** ^1^Fraunhofer Institute for Digital Medicine MEVIS, Bremen, Germany; ^2^Image-Based Biomedical Modeling, Department of Informatics, Technical University of Munich, Munich, Germany; ^3^Department of Quantitative Biomedicine, University of Zurich, Zurich, Switzerland; ^4^Department of Neuroradiology, Technical University of Munich (TUM) School of Medicine, Klinikum Rechts Der Isar, Technical University of Munich, Munich, Germany; ^5^TranslaTUM - Central Institute for Translational Cancer Research, Technical University of Munich, Munich, Germany; ^6^Helmholtz AI, Helmholtz Zentrum Munich, Munich, Germany; ^7^Athinoula A. Martinos Center for Biomedical Imaging, Massachusetts General Hospital and Harvard Medical School, Boston, MA, United States; ^8^Fraunhofer Institute for Digital Medicine MEVIS, Lübeck, Germany; ^9^Diagnostic Image Analysis Group, Radboud University Medical Center, Nijmegen, Netherlands

**Keywords:** deep learning, image registration, convolutional neural network, MRI, tumor, brain

## Abstract

Registration methods facilitate the comparison of multiparametric magnetic resonance images acquired at different stages of brain tumor treatments. Image-based registration solutions are influenced by the sequences chosen to compute the distance measure, and the lack of image correspondences due to the resection cavities and pathological tissues. Nonetheless, an evaluation of the impact of these input parameters on the registration of longitudinal data is still missing. This work evaluates the influence of multiple sequences, namely T1-weighted (T1), T2-weighted (T2), contrast enhanced T1-weighted (T1-CE), and T2 Fluid Attenuated Inversion Recovery (FLAIR), and the exclusion of the pathological tissues on the non-rigid registration of pre- and post-operative images. We here investigate two types of registration methods, an iterative approach and a convolutional neural network solution based on a 3D U-Net. We employ two test sets to compute the mean target registration error (mTRE) based on corresponding landmarks. In the first set, markers are positioned exclusively in the surroundings of the pathology. The methods employing T1-CE achieves the lowest mTREs, with a improvement up to 0.8 mm for the iterative solution. The results are higher than the baseline when using the FLAIR sequence. When excluding the pathology, lower mTREs are observable for most of the methods. In the second test set, corresponding landmarks are located in the entire brain volumes. Both solutions employing T1-CE obtain the lowest mTREs, with a decrease up to 1.16 mm for the iterative method, whereas the results worsen using the FLAIR. When excluding the pathology, an improvement is observable for the CNN method using T1-CE. Both approaches utilizing the T1-CE sequence obtain the best mTREs, whereas the FLAIR is the least informative to guide the registration process. Besides, the exclusion of pathology from the distance measure computation improves the registration of the brain tissues surrounding the tumor. Thus, this work provides the first numerical evaluation of the influence of these parameters on the registration of longitudinal magnetic resonance images, and it can be helpful for developing future algorithms.

## 1. Introduction

In neurosurgery for tumor resection, a pre-operative MRI acquisition is obtained to plan the surgical removal. After neurosurgery, MRI images are also acquired at follow-up stages to identify any pathology recurrence (Bette et al., 2017). The identification of pathological tissues in post-operative acquisitions can be improved by comparing MRI images obtained at subsequent stages of neurosurgical treatments (Verma et al., 2008). Registration algorithms are used to establish correspondences for a precise visual inspection between the subsequent MRI scans (Waldmannstetter et al., 2020). Mass effects, pathology resection, and tumor regrowth produce large deformations in the close-to-tumor regions (van der Hoorn et al., 2016). To accommodate these changes, linear registration algorithms are not accurate enough (Klein et al., 2009). Instead, non-rigid registration solutions are a better option, because they generate deformations fields that can locally register brain areas.

Several methods to register pre- and post-operative MRI images are already available. The authors in Chitphakdithai and Duncan (2010) propose a solution to register corresponding healthy tissues of longitudinal images. Furthermore, the same authors (Chitphakdithai et al., 2011) develop a method to register pre-operative MRI data with any stage of images acquired after tumor resection. By estimating missing correspondences, their algorithm encourages the accommodation of the tissues surrounding the tumor. Another solution is proposed by Han et al. (2019), in which the authors register T1 MRI images by excluding pathological tissues from the computation of the distance measure (see Equations 1 and 2 for a better explanation). Furthermore, the authors in van der Hoorn et al. (2016) propose a semi-automatic method to register pre-operative, post-operative, and follow-up images of individual patients. Their approach first semi-automatically segments brain contours, ventricles, enhanced tissues, and resection cavity in the pre- and post-operative images. In the second step, T1-CE volumes and the masks are used as input to a registration method. Besides, in Kwon et al. (2013) the authors propose a method to register pre-operative and post recurrence brain tumor images. The acquisitions are registered by excluding the pathological tissues from the image-correspondence term. T1-CE and T1 MRI sequences are used to guide the registration process. One of the few algorithms based on deep learning to register longitudinal MRI data is proposed by Lao et al. (2020). 3D T1 images are registered by excluding the segmentation of pathological tissues. A 5-level 3D U-Net model is trained on the registration of inter-patient data. In the test phase, they use volumes coming from 18 longitudinal studies, each having 2 follow-up acquisitions. Moreover, the work proposed by Estienne et al. (2020) is based on a convolutional neural network (CNN). The authors propose a joint segmentation-registration solution to (i) automatically segment pathological tissues in the moving and fixed images and (ii) register the pair of multiparametric images by excluding the automatically segmented structures from the computation of the distance measure. T1, T2, T1-CE, and FLAIR sequences are all used as input.

Multiple MRI sequences are acquired at subsequent stages of the neurosurgical treatment to better identify pathological tissues (Kwon et al., 2013; Han et al., 2019; Baheti et al., 2021). The recommended minimum requirements in neurosurgery include T1-CE, T1, T2, and FLAIR (Ellingson et al., 2015). The standard for T1-CE and T1 images is usually to acquire high-resolution 3D isotropic volumes, whereas for T2-weighted 2D acquisitions are obtained (Ellingson et al., 2015; Menze et al., 2015). Image-based registration algorithms using high-resolution images are likely to obtain better results than those utilizing lower resolution images, such as FLAIR and T2w acquisitions. Nevertheless, the already proposed solutions utilize different MRI protocols to guide the registration process. A numerical evaluation of the influence of multiple sequences on the registration of longitudinal MRI data is still missing.

Furthermore, image-based registration algorithms rely on the fact that corresponding structures can be found in the pairs of images to be registered. This assumption is not valid for the registration of longitudinal MRI acquisitions acquired during tumor resection. The pathological tissues visible in pre-operative acquisitions are removed, and are not observable in post-removal images. Many of the proposed registration methods tackle this problem by excluding the contribution of pathological tissues from the correspondences computation (Chitphakdithai et al., 2011; Kwon et al., 2013; Han et al., 2019). In fact, it is commonly assumed that the outcome of registration process improves if only corresponding (healthy) structures are taken into account. However, no exhaustive evaluation of influence of the exclusion of the pathological tissues has been done yet.

First, this work aims to evaluate the influence of different MRI sequences on the registration of longitudinal MRI data. To the best of our knowledge, it is the first time that this analysis is performed. More details are given in Section 2.3.1. Second, this work quantitatively analyzes the effects of excluding (and including) the pathological tissues from the computation of distance measure used for registration of longitudinal MRI data (more details in Section 2.3.2). Two registration approaches are proposed for performing the aforementioned experiments, an iterative method, and a CNN-based solution. The exclusion of the pathological tissues in the CNN method is performed only during training, whereas the iterative method excludes them during the registration process.

## 2. Materials and methods

### 2.1. Datasets

For the volume of each dataset, four different MRI sequences are available: native T1, T1-CE, T2 and FLAIR. Each case is normalized using the same preprocessing (Menze et al., 2015): Every volume is skull-stripped, noise corrected, rigidly registered to an atlas reference volume, and interpolated to 1*mm*^3^ voxel resolution. The images have a size of 240 × 240 × 155 and are downsampled to 160 × 160 × 160 to be input to the registration methods.

#### 2.1.1. Munich dataset

This set includes two or more consecutive post-operative acquisitions of 66 patients, acquired at the Klinikum Rechts der Isar during 2015 and 2020 (Paprottka et al., 2021). From this dataset, we choose a subset of 57 patients to only include volumes characterized by four MRI sequences. The original acquisitions for each patient include an isotropic T1 (voxel size of 1 mm^3^) before and after contrast, axial T2 (voxel size of 0.72 × 0.72 mm^2^), an isotropic FLAIR (voxel size 1 mm^3^). The volumes are available after normalization performed according to Menze et al. (2015). The pathological tissues are automatically segmented (Paprottka et al., 2021). To generate a robust brain tumor segmentation, we use an iterative process. First, we generate binary segmentation masks using five segmentation algorithms (Feng et al., 2019; Isensee et al., 2019; McKinley et al., 2019, 2020; Zhao et al., 2019) developed within the scope of the BraTS challenge (Menze et al., 2015; Bakas et al., 2017a,b, 2018) using BraTS Toolkit *btk*) (Kofler et al., 2020). Second, we fuse the segmentation masks using equally weighted majority *via btk* (Kofler et al., 2020). Third, a visual inspection is conducted to correct the fused segmentation masks. This approach promises to achieve a higher segmentation quality than a pure manual delineation (Kofler et al., 2021) while saving valuable expert radiologists' time.

#### 2.1.2. BraTS 2015: Validation set

BraTS 2015 dataset includes a mixture of pre-operative and follow-up MRI images. A subset of the BraTS 2015 training dataset is chosen (Menze et al., 2015; Bakas et al., 2017b, 2018; BraTS, 2021), to include only longitudinal studies. This subset includes 45 pairs of images, each composed of a pre- and a post-operative acquisitions. According to Menze et al. (2015), the original acquisitions for the images sets include a T1 image (1–6 mm slice thickness), a T1-CE image (voxel size of 1 mm^3^ for most patients), a T2 image (with 2–6 mm slice thickness), a FLAIR image (2–6 mm slice thickness). The ground truth masks of the pathological tissues are also available. Moreover, the resection cavity, not originally segmented in the original ground truth, has been manually segmented in a previous work (Canalini et al., 2021).

#### 2.1.3. BraTS 2015: Test set

**Figure 1:**
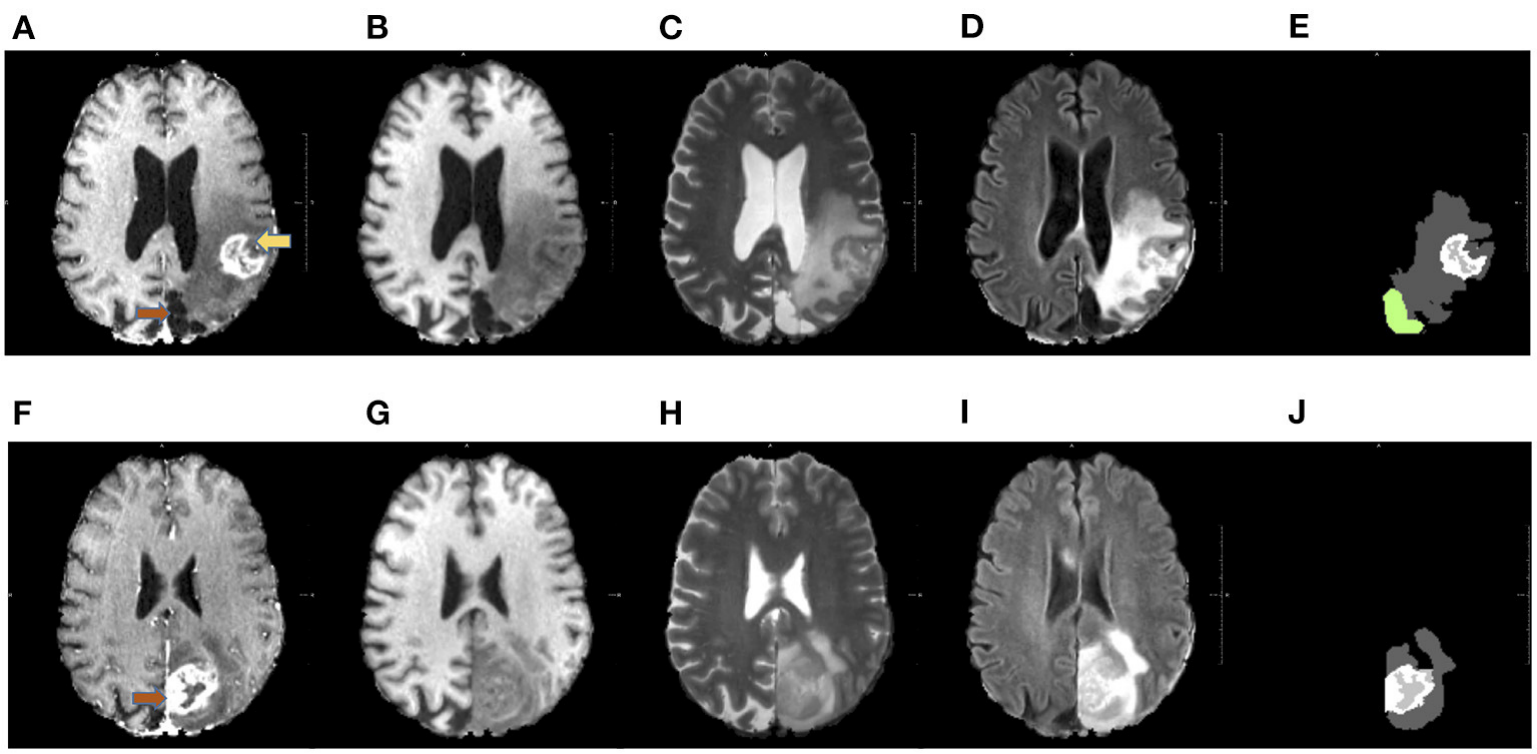
Available MRI sequences. The figures in the first row show example slices of a post-operative acquisition, whereas in the second row images of the corresponding pre-operative volume are displayed. Each volume comprehends four sequences: T1 gadolinium contrast-enhanced T1-CE in **(A,F)**, T1 in **(B,G)**, T2 **(C,H)**, and FLAIR in **(D,I)**. Each sequence is useful to spot a particular component of the pathological tissues. For example, in T1-CE the enhanced tissue is observable, whereas on FLAIR sequence the edema is well visible. The masks of the pathological tissues available for this work are visible in panels **(E,J)**. In **(E)**, the resection cavity is colored green. The pre-operative tumor and the corresponding resection cavity are indicated by the orange arrows. In after surgery acquisitions, pathological tissues can also be present, as in this example (the post-operative tumor is pointed by the yellow arrow).

**Figure 2:**
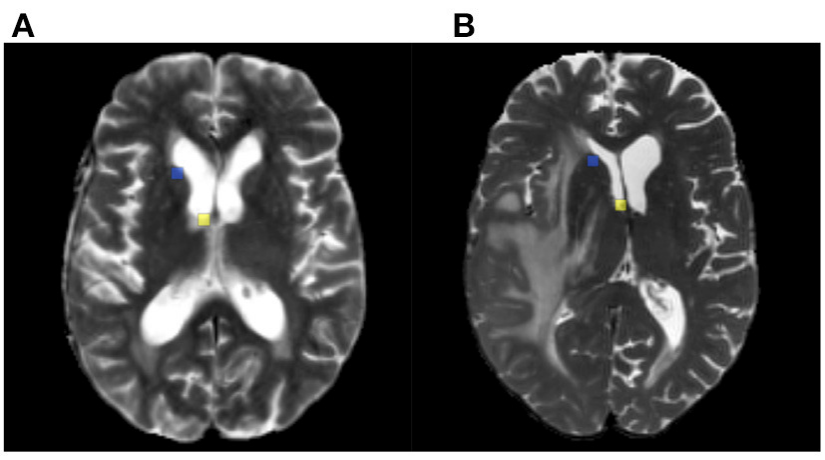
Example of annotated landmarks on test set. Panels **(A,B)** correspond to post and pre-operative MRI acquisitions of the same patient. The corresponding landmarks are visualized with the same colors.

A subset of the BraTS 2015 test set is selected, to include only longitudinal studies. It has 59 pairs of images of the different patients. The acquisition details of this dataset are the same described in the previous subsection (Section 2.1.2) (Menze et al., 2015). The masks of the pathological tissues (edema, enhancing tumor, necrosis non-enhancing tumor) have been already segmented in the original dataset. Moreover, the resection cavities in the post-operative volumes are manually segmented by two raters. An example of the finally available structures is shown in the fifth column of Figure 1. To compute the registration results, six landmarks have been manually acquired for each pair of longitudinal acquisitions. First, for each pre-operative scan, landmarks are acquired near the tumor (within 40 mm). Second, corresponding markers are obtained in post-operative images. The landmarks are acquired on anatomical structures, such as brain sulci and gyri, and the midlines of the brain. An example of the annotated landmarks is available in Figure 2. One or two raters annotated them and an experienced neuroradiologist evaluated them clinically. The baseline mean target registration error (mTRE) 2.92 mm.

#### 2.1.4. BraTS-Reg challenge dataset

**Figure 3:**
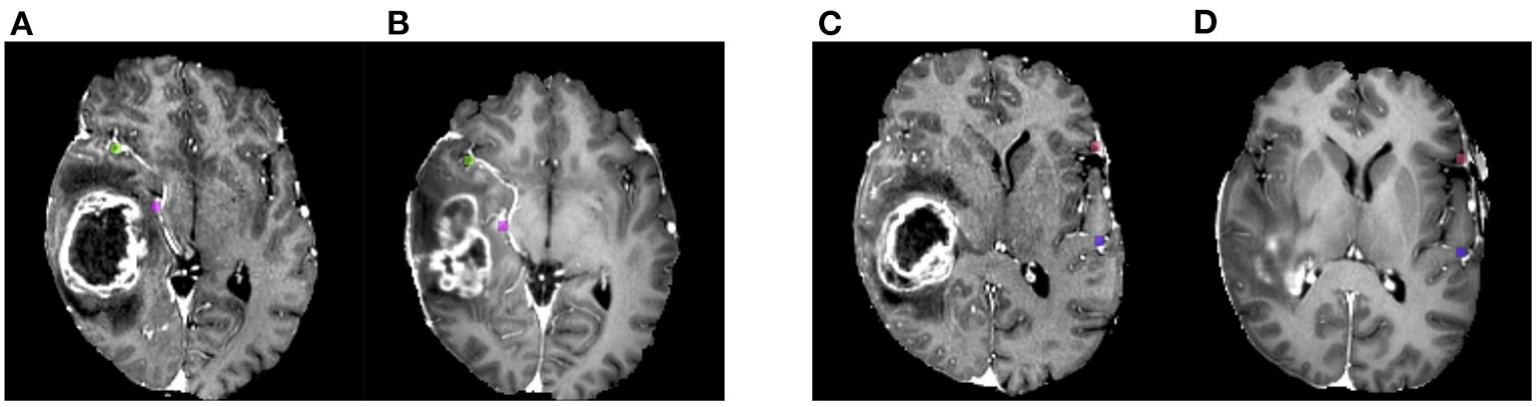
Example of annotated landmarks on BraTS-Reg dataset. Panels **(A,C)** show different axial images of the post-operative acquisition for the same patient, whereas **(B,D)** correspond to the corresponding locations in the pre-operative image. The corresponding landmarks are visualized with the same colors.

The dataset includes 140 pairs of pre and post-operative MRI volumes (Baheti et al., 2021). The time-window between all pairs of pre-operative and follow-up volumes is in the range of 27 days–37 months. This dataset comprises already pre-processed image sets collected in affiliated and public institutions. Although no information about the acquisition parameters is provided by the challenge organizers, it is likely to assume that the data have been acquired following the standard protocols mentioned in Menze et al. (2015) and already described in Section 2.1.2. Several raters manually annotated 6–50 corresponding landmarks between the pre- and post-operative volumes. For each pre-operative scan, landmarks are acquired near the tumor (within 30 mm) and far from the tumor (beyond 30 mm). Thus, matching points are obtained in post-operative images. The landmarks are anatomical structures, such as blood vessel bifurcations, the anatomical shape of the cortex, and anatomical landmarks of the midline of the brain (Baheti et al., 2021). An example of the annotated landmarks in BraTS-Reg is available in Figure 3. After a rigid pre-registration between the pre- and post-operative volumes, the baseline mTRE is 3.62 mm. The automatic algorithm already applied for Section 2.1.1 is here utilized to segment the pathological structures in every volume of this dataset.

### 2.2. Methods

This work investigates two different types of registration methods, an iterative solution and a CNN-based approach. The following design concepts are valid for both approaches.

The reference (post-operative) and template (pre-operative) images can be modeled as functions R, T : ℝ^3^ → ℝ. The goal of the proposed image registration approaches is to generate a deformation y:Ω → ℝ^3^ that aligns the two images R and T on the field of view Ω⊂ℝ^3^ such that R(x) and T(y(x)) are similar for x∈Ω. The deformation field represents a minimizer of the cost function:


(1) J(ℛ,T,y)=D(ℛ,T(y))+αR(y)

The first term D is a distance measure computing the difference between the reference R and the warped template image T(y) summing pointwise distances. A challenge of registering longitudinal MRI data is that a one-to-one correspondence between the two images is not guaranteed due to the resection of pathological tissues. We tackle the problem by adding the possibility of excluding the contribution of the pathology of the fixed image from the computation of the distance measure. By indicating the pathological tissues as Σ, the distance measure is computed as follows:


(2) $$D(ℛ,T(\text{y}))=\int_{\Omega \backslash \Sigma }d(ℛ(x),T(\text{y}(x))\text{ d}x$$

In our settings, R and T, respectively, refer to the post-operative and pre-operative volumes. Thus, Σ corresponds to the pathological masks of the post-operative image. More details about the distance measure are available in Equation (3).

The second term *R* in Equation (1) is the regularizer, that limits the possible deformations that can be computed during the minimization process. The hyperparameter α controls the strength of the minimization term. More details about the regularization chosen in this work are available in Equations (4) and (5).

#### 2.2.1. Multi-level deep learning method

The deep learning registration method used in this work is based on the solution proposed by Hering et al. (2021). It is a multi-level variational image registration approaches, that combines deformation fields computed at different image scales. Since the solution already achieved good results in other medical imaging fields, we want to evaluate how this method perform on longitudinal MRI data.

The solution has been originally proposed to register lung data, where also corresponding masks and landmarks were available to compute additional similarity terms. In our case, only intensity brain volumes are available. Therefore, we use only the intensity images to compute the similarity. Due to the lack of consistent intensity profiles in the MRI acquisitions (Baheti et al., 2021), and the presence of pathological tissues, the normalized gradient fields (NGF) measure is chosen as distance metric (Haber and Modersitzki, 2006). The use of the NGF is based on the observation that two images are considered similar if intensity changes occur at the same locations. Instead of computing the magnitude of the image gradient (∇R(x) and ∇T(x), respectively, for the reference and template image), the normalized gradient field is utilized (Haber and Modersitzki, 2006). The goal of image registration based on NGF is to align them by reducing the difference between the normalized gradient fields computed for the reference and the template image. It is defined as follows,


(3) $$\text{NGF}(ℛ,T)=\frac{1}{2}\int_{\Omega }^{}1-{\left(\frac{{\langle \nabla ℛ(x),\nabla T(x)\rangle }_{\varepsilon_{R}\varepsilon_{T}}}{\|\nabla T(x){\|}_{\varepsilon_{T}}\|\nabla ℛ(x){\|}_{\varepsilon_{R}}}\right)}^{2}dx$$

where ${\langle x,\text{y}\rangle }_{\varepsilon }:=x^{⊤}\text{y}+\varepsilon$, $||x|{|}_{\varepsilon }:=\sqrt{{\langle x,x\rangle }_{\varepsilon^{2}}}$ and ε_*R*_, ε_*T*_>0 are the so-called edge-parameters controlling influence of noise in the images. Their value has been empirically chosen. Moreover, we modified the original CNN implementation by introducing the possibility of using masks of the pathological tissues as external input during the training and validation phases, to exclude their contribution from the correspondence computation (see Equation 2). In the test phase, no mask of the pathological tissues is needed. Moreover, we also added the possibility of using input images characterized by two MRI sequences, whereas the original implementation accepted only one-channel images.

When searching for the best solution to the minimization term, multiple solutions may exist. However, not all the possible minima of the objective function represent good and realistic registration solutions. The *R* term in the objective function (see Equation 1) favors a smoother deformation field y. As in the original architecture, we utilize the curvature regularizer,


(4) $$R(\text{y})=\int_{\Omega }\sum_{k=1}^{3}\|\Delta {\text{y}}_{\text{k}}(x){\|}^{2}dx$$

which penalizes deformation fields having too large second derivatives. To limit even further the viable solutions, another regularization term


(5) $$V(\text{y})=\int_{\Omega }^{}\psi (\det\nabla \text{y}(x))dx$$

is added to the objective function, where ψ(*t*) = (*t*−1)^2^/*t* for *t*>0 and ψ(*t*): = ∞ for *t* ≤ 0. The volume change control is used to discourage foldings in the deformation field y that may be generated during the minimization of the cost function. Folding in the deformation field represents an unrealistic transformation that the minimization process may lead to. The hyperparameter controlling the influence of this extra term on the loss function is γ, thus the final term to be added is γV(y).

The proposed solution is based on a 3D U-Net architecture that takes as input the concatenation of the 3D fixed (follow-up) and the moving (pre-operative) image and provides as output a 3D dense deformation field with a resolution identical to the images (Hering et al., 2019b). The following description about the CNN architecture is based on what reported in Hering et al. (2019b). The network consists of three levels starting with 16 filters in the first layer, doubled after each downsampling step. 3D convolutions are used in both encoder and decoder path with a kernel size of 3 followed by an instance normalization and a ReLU layer. In the encoder path, the feature map downsampling steps use average pooling with a stride of 2. In the decoder path, the upsampling steps use transposed convolution with filters and half the number of filters than the previous step. The final layer uses a 1 × 1 × 1 convolution filter to map each 16-component feature vector to a three-dimensional displacement.

The datasets (Sections 2.1.2, 2.1.1) are used as training set. Each CNN model is trained for 40 epochs. The loss weighting parameters are set as follows: α = 0.1, γ = 0.01. The learning rate is set to 0.001. The values of these parameters are different from the original implementation and were empirically modified. Besides using individual sequences, input characterized by two features (i.e., two MRI sequences) are also utilized to train the CNN method. The combinations of MRI sequences used to train and test the CNN solution are available in **Table 2**. The use of data input characterized by two features is a novelty with respect to the original architecture, where only one channel images have been used (Hering et al., 2019b).

The registration is performed on three levels (*L* = 3) by using images at different scales. The deformation field is initially computed on the coarsest level and the images are downsampled by a factor equal to 2^L-1^. On a finer level, the previously computed deformation fields are utilized as an initial guess by warping the moving image. At each level, the moving and fixed images are downsampled. Due to graphical memory issues, the finest resolution of the registered images is 160 × 160 × 160, which is also the size of the generated deformation field (Hering et al., 2019b). The final deformation field is then upsampled to the original size of the input images.

#### 2.2.2. Multi-level iterative method

The iterative solution utilized in this work is a variational image registration approach (Modersitzki, 2009). This method has been already used in neurosurgical context (Canalini et al., 2019) and, in this work, we evaluate it on longitudinal MRI data. The registration can be considered as an iterative optimization algorithm where the search of the correct registration between two images corresponds to an optimization process aimed at finding a global minimum of an objective function. The objective function has to be minimized for each image pair and the minimization process is performed according to a discretize-then-optimize paradigm. The objective function to be minimized includes a distance measure, quantifying the similarity between the warped template image and the reference one, and a regularizer, which favors the smoothness of the computed deformation fields. NGF is here used as a distance measure (see Equation 3), and a curvature regularizer is utilized (see Equation 4). The method also allows to mask the pathological tissues out from distance measure computation (see Equation 2). However, differently from the CNN method, these segmentations are needed in the test phase. Moreover, in the iterative method, the choice of the optimal transformation parameters is conducted by using the quasi-Newton l-BGFS (Liu and Nocedal, 1989), due to its speed and memory efficiency.

The iterative method performs a non-parametric registration that, as for the deep learning method, the is performed on three levels (*L* = 3) by always using images at different scales. On the finest level, the volumes have a size of 160 × 160 × 160. The deformation field obtained in output from the iterative method is then upsampled to the size of the original images. The stopping criteria for the optimization process are empirically defined: the minimal progress, the minimal gradient, and the relative one, the minimum step length are set to 0.001, and the maximum number of iterations is set to 100. The loss weighting parameter is empirically set to α = 0.1. The registration algorithm is used to register the volumes of the test set.

#### 2.2.3. Diffeomorphic registration ANTs method

The Symmetric Diffeomorphic registration method ANTs represents a standard registration algorithm for MRI data (Avants et al., 2008; Antsx, 2009). Thus, we aim to evaluate how well the proposed methods perform in comparison to a standard approach. ANTs is applied by using the original size of the volumes (240 × 240 × 155). As suggested in Ou et al. (2014), the Symmetric Normalization (SyN) transformation model of ANTs is utilized, and cross-correlation is used as the distance measure, since NGF is not available. The T1-CE is used to guide the registration, since it is supposed to be the sequence with the higher original resolution and, thus, the one leading to better registration results. Moreover, the masking of the distance measure is performed, to reduce the negative effects of the non-corresponding tissues on the registration results.

### 2.3. Experiments

#### 2.3.1. Influence of different MRI sequences

This work aims to numerically analyze the influence of multiple MRI sequences on the registration of longitudinal data by evaluating the performances of two types of method, the iterative solution and the deep learning-based approach. The CNN method is trained on four individual sequences (T1-CE, T1, T2, and FLAIR). In the inference process, the models are then used to register the corresponding sequences in the test sets. Besides, the iterative method is applied to different individual sequences. Moreover, we also train the CNN solution using input volumes characterized by two distinct sequences. This experiment aims to verify whether multiple MRI sequence input is better than only one to train the neural network models. The deformation field computed on an individual or multiple sequences can then be applied to the other MRI acquisitions of the same patient.

The non-parametric Wilcoxon signed-rank test is utilized to verify whether there is a statistically significant difference between the results of each registration solution (iterative method and trained CNN models) and the baselines of the two test sets. This analysis tests whether the median of the differences of the two paired results is zero. The data distribution of the baseline registration errors is not normal according to the One-sample Kolmogorov-Smirnov test (Pettitt and Stephens, 2012).

#### 2.3.2. Effects of excluding the pathological tissues from the distance measure

This work also evaluates the influence of the pathology on the registration process. Thus, masks are used to exclude the contribution of the pathological tissues from the distance measure computation. For the CNN method, the segmentation of the pathological tissues is used as extra input only during training, to compute the distance measure on the healthy tissues (see Equation 2). In the inference process, no mask is required. Four additional models are trained, each for a different MRI sequence, without masking the distance measure. The iterative method also has the possibility of excluding the pathological tissues from the distance measure. However, it requires the segmentation of pathological tissues when applied to the test set. The iterative method excluding and including the pathological tissues is also utilized for each of the four MRI sequences.

## 3. Results

The mean target registration errors are computed to evaluate the outcome of the different registration algorithms. For each matching landmark (see Figure 2 for an example), the Euclidean distance between its position in the reference image and its position in the moving image is computed. Then, for each patient set, the mean Euclidean distance among all the landmarks is calculated. Thus, the mean value of the distances of all the image sets of a test dataset (i.e., the mTRE) is estimated. The proposed methods output the warped moving image and the deformation field. The latter is applied to the landmarks and, if the value obtained after registration is lower than the initial baseline, the warped moving images is supposed to be better registered to the corresponding reference images. The results shown in this paper are computed on two different test sets (Sections 2.1.3, 2.1.4).

**Figure 4:**
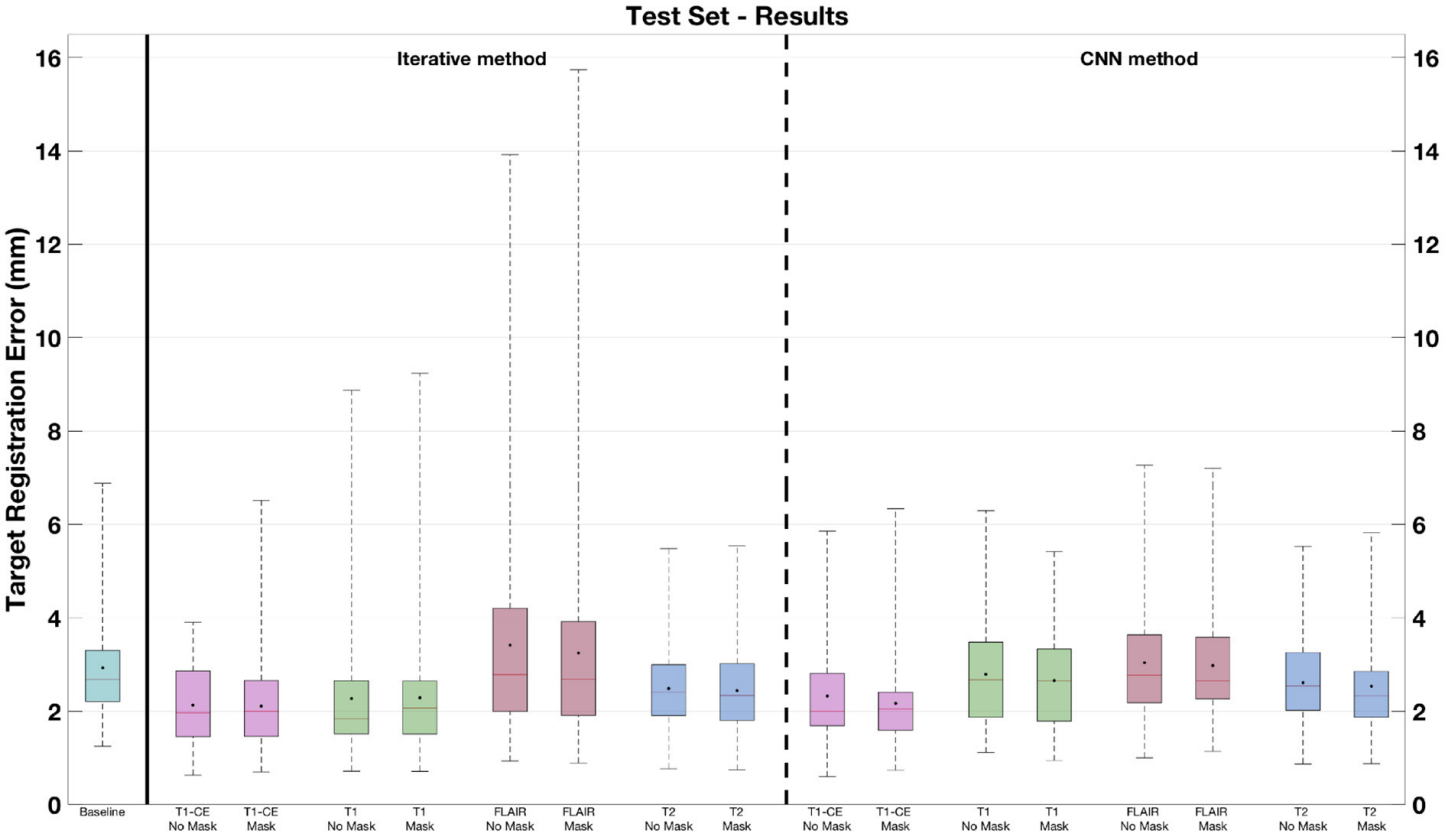
Target registration errors on the test set. The box plots related to the solutions where no masking procedure is performed are indicated as *No Mask*. In each box plot, the red line and the point, respectively, indicate the median and mean values.

**Figure 5:**
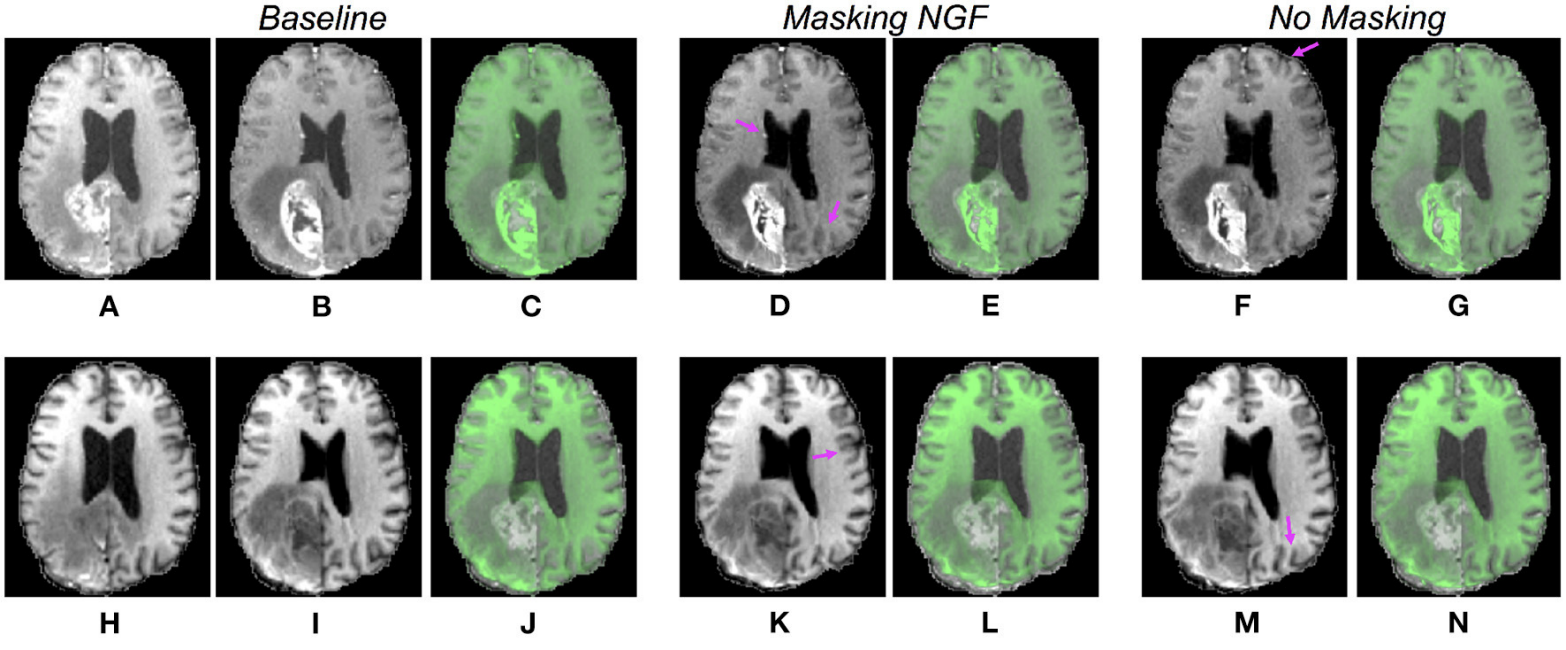
Comparison of qualitative results for CNN models trained on T1-CE and T1 MRI sequences. Each row refers to the results for the CNN models trained on different sequences (from the top to the bottom, T1-CE and T1). The post- and pre-operative images are visualized in the first and second columns **(A,B,H,I)**, and the initial overlay between the two acquisitions is visible in the third column **(C,J)**. The last four columns display the warped moving volumes **(D,F,K,M)** and the overlays between the fixed image (post-operative) and the warped moving images **(E,G,L,N)**, respectively, for the models excluding and including the pathology in the distance measure. The purple arrows point to locations where improvements are observable. In panels **(D,K)**, a better overlap of the lateral ventricles is visible. Moreover, in panels **(F,K,D)** the sulci are better registered.

**Figure 6:**
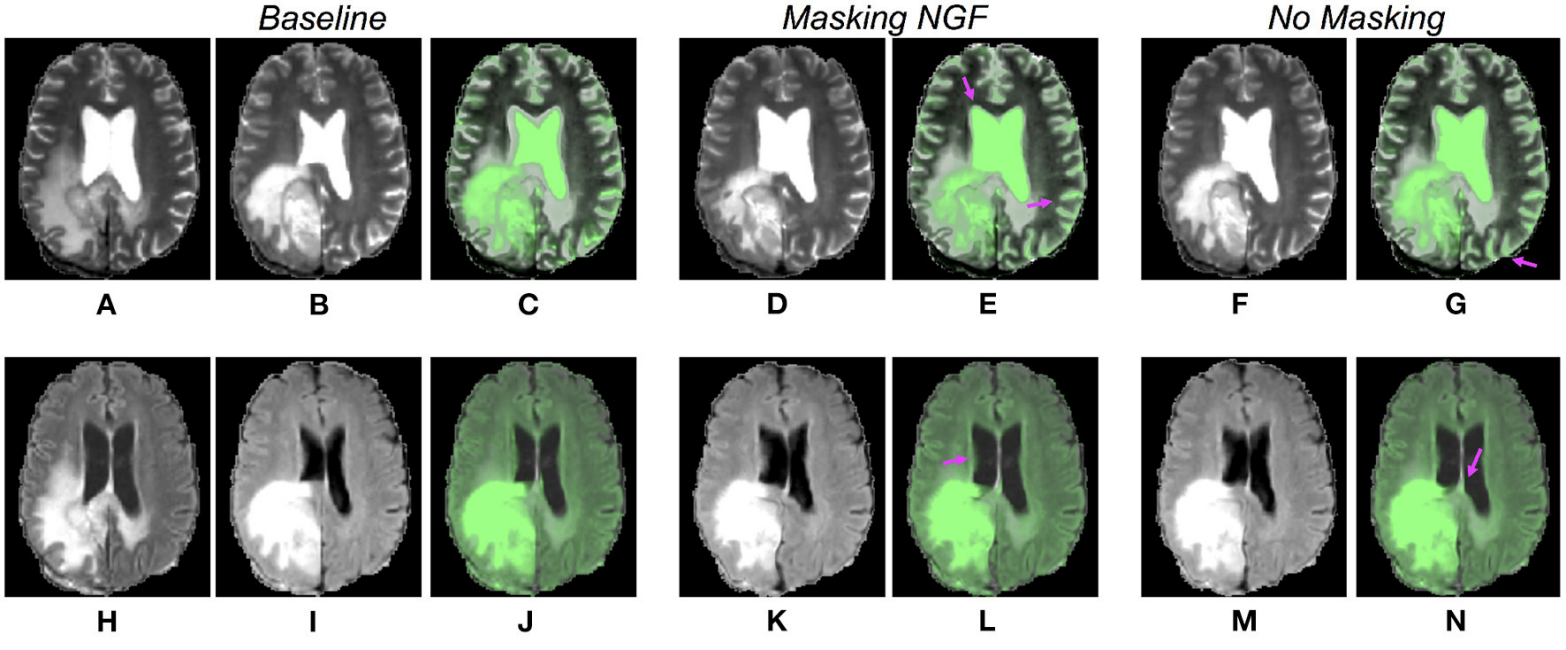
Comparison of qualitative results for CNN models trained on T2 and FLAIR MRI sequences. Each row refers to the results for the CNN models trained on different sequences (from the top to the bottom, T2 and FLAIR). The post- and pre-operative images are visualized in the first and second columns **(A,B,H,I)**, and the initial overlay between the two cases in the third column **(C,J)**. The last four columns display the warped moving volumes **(D,F,K,M)** and the overlays between the fixed image (post-operative) and the warped moving images **(E,G,L,N)**, respectively, for the models excluding and including the pathological tissues in the distance measure. The purple arrows point to locations where improvements are observable. In panels **(E,L,N)**, a better registration of the lateral ventricles is visible. Besides, in panels **(G,E)**, the sulci are more aligned.

**Figure 7:**
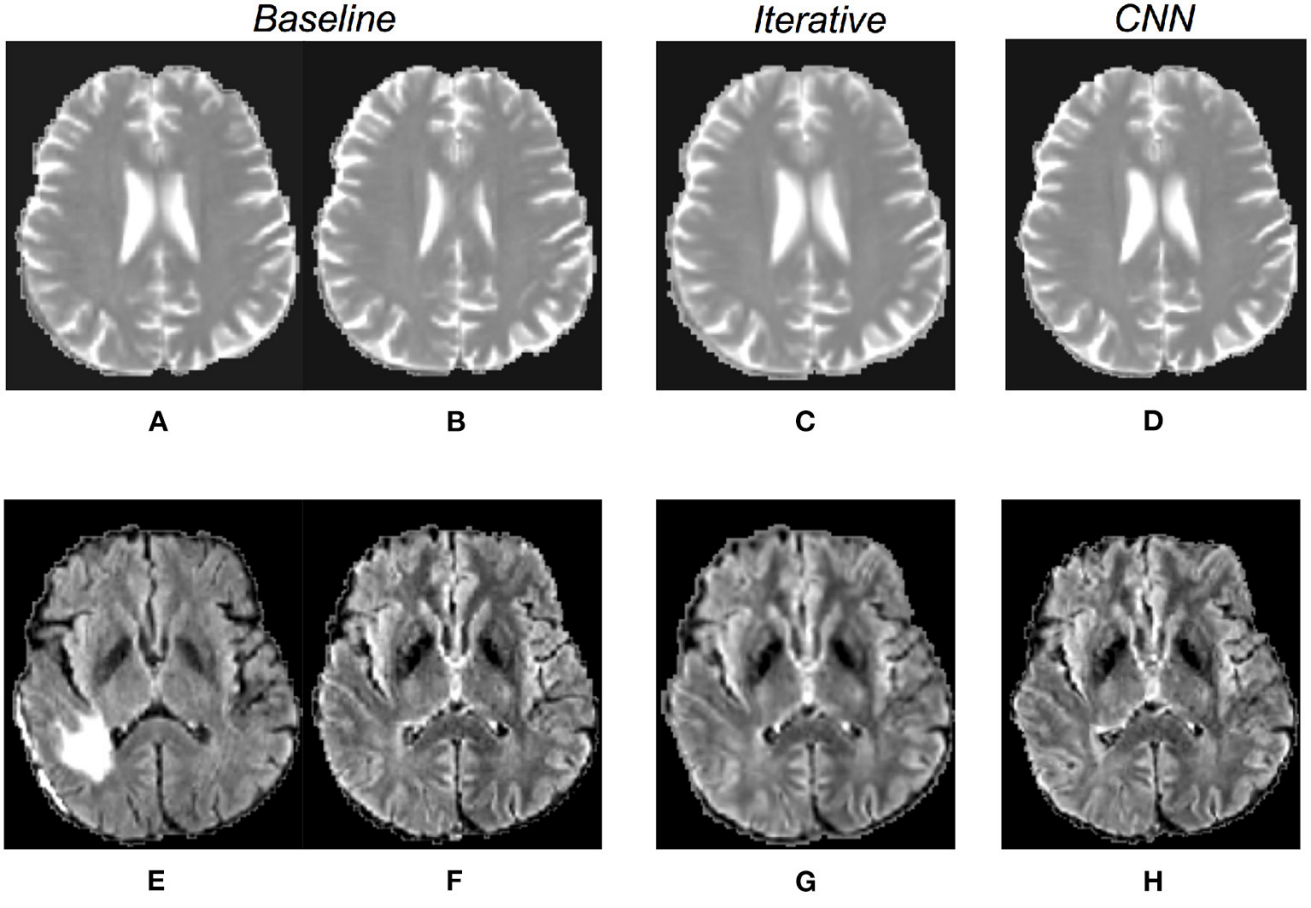
Qualitative results obtained by CNN and iterative solutions masking the distance measure. The first row is related to methods using the T2 MRI sequence, whereas the last one shows example results for solutions using the FLAIR sequence. The first two columns show the corresponding slices of the pre- and post-operative volumes **(A,B,E,F)**, rigidly registered in the pre-processing step. The third column presents warped moving images obtained by the iterative method **(C,G)**, the fourth column shows the results for the CNN models **(D,H)**.

Table 1 shows the mTREs obtained by the proposed solutions. For the CNN models trained without masking, the lowest mTRE (2.32 mm) is obtained by the model using the T1-CE, whereas the highest value is achieved when the FLAIR sequence is used (3.04 mm). Besides, the iterative method using this sequence also achieves the highest mTRE (3.41 mm). The lowest mTRE is obtained with the T1-CE (2.13 mm). A further comparison of the results obtained on different MRI sequences by the two methods is visible in Figure 4. In both cases, the FLAIR sequence leads to higher median TREs, with a large range of results. On the contrary, the T1-CE and T2 sequences help to lower the median TREs and limit the ranges of values. Besides, the Wilcoxon test is utilized considering the target registration errors. In both methods, the null hypothesis cannot be accepted for the models trained on T1-CE (*p* < 0.000001), and on T2 (*p* < 0.01). Figure 5 shows the qualitative results of CNN models trained on T1-CE and T1 MRI sequences, and Figure 6 those of the methods using the T2 and FLAIR MRI sequences. The qualitative results for the iterative method using T2 and FLAIR MRI are visible in the third column of Figure 7. In this figure, the visual results of the iterative method using T2 or FLAIR are provided.

**Table 1:** Mean target registration errors for the test set.

	**mTRE (mm)**			
Baseline	2.92			
ANTs (T1-CE, mask)	2.37			
MRI sequence	CNN	CNN mask	Iterative	Iterative mask
T1	2.79	2.65	2.27	2.29
T1-CE	2.32	2.16	2.13	2.11
FLAIR	3.04	2.98	3.41	3.24
T2	2.61	2.53	2.48	2.44

The mean TREs obtained by CNN models trained with and without masking procedure are visible in the second and third column. The results achieved by the iterative method in the fourth and fifth column.

Moreover, for what concerns the results obtained by discarding the contribution of the pathology, Table 1 shows that the lowest and highest mTREs obtained by the CNN without masking the pathological are reduced when these tissues are excluded from the distance measure computation (respectively, 2.16 and 2.98 mm). When the tumor is excluded, an mTRE of 3.24 mm is achieved by the iterative method using FLAIR. The lowest mTRE in our experiments is obtained when registering the T1-CE MRI sequence (2.11 mm). A comparison between each method excluding or not the pathological tissues from the distance measure is also available in Figure 4. Besides, the fourth column in Figure 7 shows the registration results for two CNN models trained on longitudinal data by excluding the pathological tissues from the distance measure. Furthermore, in Table 2, the lowest mTRE (2.41 mm) is obtained by CNN trained with T1-CE and T2 sequences. On the contrary, the highest mTRE of 2.82 mm is achieved by the solution using FLAIR and T2 sequences.

**Table 2:** Mean target registration errors of the multisequence trained models on the test set (first table) and BraTS-Reg dataset (second table).

**mTRE (mm) - Test set**		**mTRE (mm) - BraTS-Reg set**	
**Sequence**	**CNN mask**	**Sequence**	**CNN Mask**
T1-CE + T2	2.41	T1-CE + T2	2.94
T1-CE + T1	2.45	T1-CE + T1	3.17
T1 + T2	2.48	T1 + T2	3.58
T1-CE + FLAIR	2.43	T1-CE + FLAIR	3.15
T1 + FLAIR	2.61	T1 + FLAIR	3.23
FLAIR + T2	2.82	FLAIR + T2	3.03

**Figure 8:**
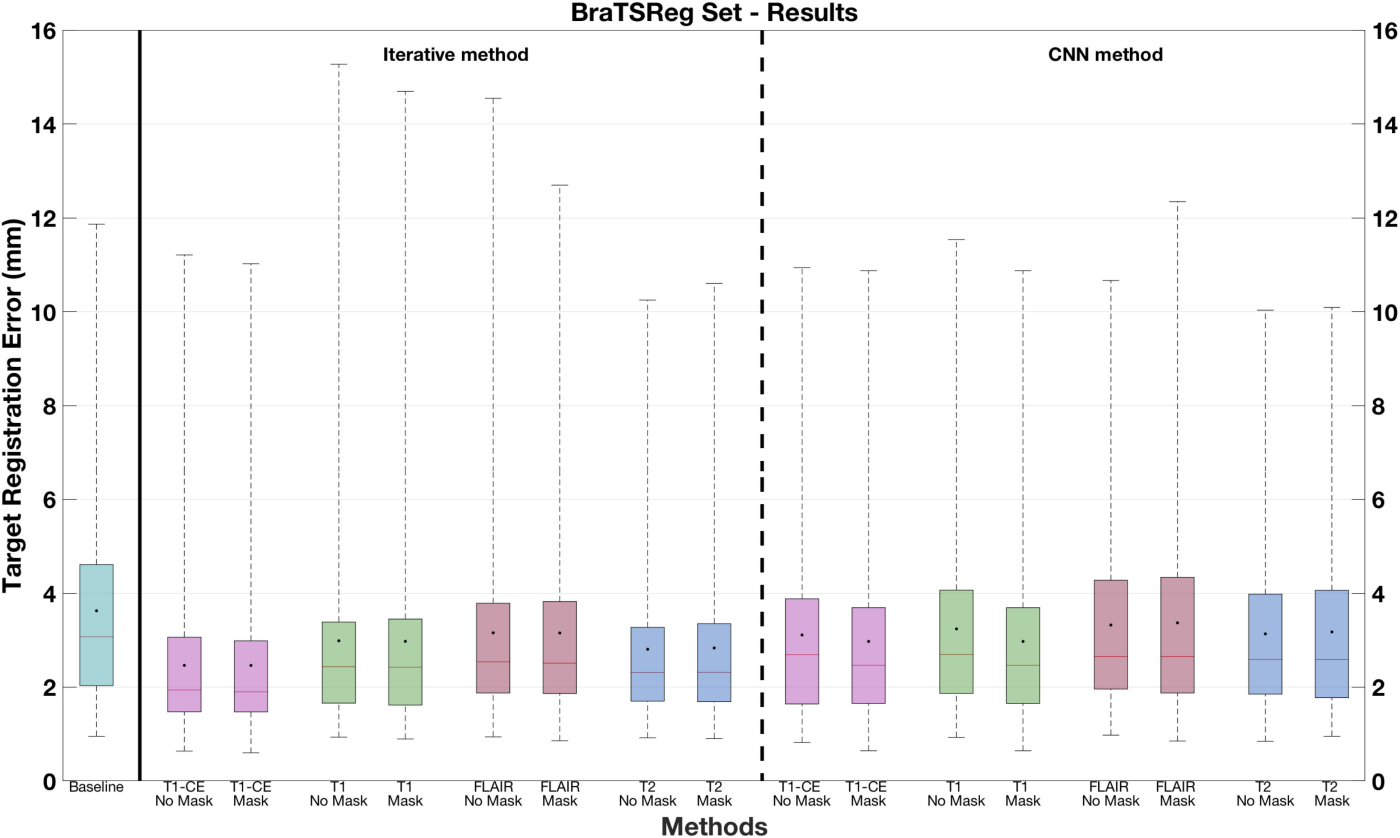
Target registration errors on the BraTS-Reg dataset. The box plots related to the solutions where no masking procedure is performed are indicated as *No Mask*. In each box plot, the red line and the point, respectively, indicate the median and mean values.

The CNN models trained on individual sequences, as well the iterative methods, are also applied to the BraTS-Reg dataset. The mTRE results are available in Table 3 and Figure 8. The results of both methods are lower when using the T1-CE and the T2 sequences, whereas the highest mTREs are achieved on the FLAIR sequence. Figure 8 shows that, when using FLAIR sequence, both methods lead to a range of values even higher than the baseline. Moreover, when the iterative method uses the T1 sequence, some cases also have larger TRE than before registration. On the contrary, when using T2 and T1-CE sequences, smaller ranges of values are achieved. When comparing the CNN models masking the pathological tissues and those not excluding them in Table 3, we can observe a lower value only for the networks trained on T1-CE. However, higher mTREs are obtained by the CNN methods trained on FLAIR and T2. Besides, almost no difference can be seen between the sections related to the traditional method (Iterative vs. Iterative masks). A more detailed overview is observable in Figure 8, comparing for each sequence the box plots labeled as *No Mask* and *Mask*. Besides, the second section of Table 2 provides the results obtained on the BraTS-reg dataset by the CNN models trained on multiparametric input. The numerical result obtained by using T1-CE and T2 achieves the lowest mTRE obtained by the CNN method on the BraTS-Reg dataset.

**Table 3:** Mean target registration errors for the BraTS-Reg Dataset.

	**mTRE (mm)**			
Baseline	3.62			
ANTs (T1-CE, mask)	2.84			
MRI sequence	CNN	CNN mask	Iterative	Iterative mask
T1	3.24	3.24	2.99	2.98
T1-CE	3.11	2.97	2.46	2.46
FLAIR	3.32	3.37	3.16	3.15
T2	3.13	3.17	2.81	2.83

The mean TREs obtained by CNN models trained with and without masking procedure are visible in the second and third column. The results achieved by the iterative method in the fourth and fifth column.

According to our experiments, the T1-CE sequence and the masking of the pathological tissues from the distance measure lead to the lowest mTREs. The ANTs algorithm also uses these settings (see Section 2.2.3) and the mTRE obtained by this method on the BraTS-Reg dataset is 2.84 mm, whereas on the Test set the final value is equal to 2.37 mm.

## 4. Discussion

None of the already proposed methods analyzed the influence of different sequences on the registration of longitudinal MRI data. However, our experiments show that the choice of the MRI sequence has a strong impact on the outcome of the registration of longitudinal data. This is evident by analyzing the results obtained by two different types of registration methods, namely an iterative and CNN method, proposed for the task of non-rigidly registering longitudinal MRI data. Our experiments show that the T1-CE sequence is the best choice to design the registration algorithms, leading to better mTREs. This outcome could be explained by the fact that T1-CE images used in neurosurgery usually have a higher resolution than other MRI protocols. Moreover, thanks to the contrast enhancement, the better image contrast of anatomical tissues in this protocol could also be responsible for better registration results. On the contrary, the FLAIR is the worst to guide the registration process: This couldn't be predicted from the original acquisition parameters of the test sets, since the T1-weighted, T2 and FLAIR images are acquired with comparable resolution. These findings are also visible in Figures 4, 8, where the boxplots related to FLAIR sequence present higher median and mean TREs than the other sequences, and range of values higher than the baseline. Besides, all the multi-sequences trained CNN models improve the baseline mTRE of the test set, but none leads to an improvement in terms of registration accuracy. The deformation fields computed on this sequence can then be employed to warp the other acquisitions characterized by the other MRI protocols. Besides, the model trained on T1-CE and T2 sequences outperforms that method trained solely on T1-CE in the BraTS-Reg dataset (refer to Tables 2, 3).

Our experiments also shows that computing the distance measure on non-corresponding elements negatively impacts the registration of the longitudinal MRI data. Yet, the influence of the masking procedure differently affects the brain tissues, depending on their positions relatively to the pathological tissues. The exclusion of the pathology from the computation of the distance measure has a positive effect on the mTREs of Table 1. However, the exclusion of these tissues has almost no influence on the registration of the BraTS-Reg dataset, as shown in Table 3, except for the CNN trained on the T1-CE. where landmarks are also positioned far away from the pathology.

Furthermore, the iterative solution using downsampled volumes outperforms the CNN approach, and the standard method ANTs, that utilizes original size images in both test sets. In fact, the best improvement is obtained by the iterative method using T1-CE on the BraTS-Reg dataset, where the initial mTRE is reduced by 1.16 mm. In the test set, the initial value is reduced of 0.81 mm by this method. Instead, the CNN approach is outperformed by the standard method in the BraTS-Reg set. Besides, in the test set, the CNN method achieves better results than the standard solution.The CNN method trained on the T1-CE sequence obtains an improvement of 0.65 mm on the BraTS-Reg set, and one of 0.76 mm on the test set.

### 4.1. Limitations

The iterative method is not affected by memory issues as the CNN soluton. Thus, original resolution images could be utilized to validate the iterative method. Nevertheless, this method has been evaluated by using the resampled volumes as input, which could be suboptimal for the accuracy outcome. Although it is not uncommon to use lower resolution images to speed up the registration process, an improvement in the registration results might be achieved by using original size data. Nevertheless, the iterative solution using downsampled volumes already achieves the best results in our experiments.

Due to memory issues, the input data to the CNN had to be downsampled. By reducing the input size, the information stored in the original images gets lost. Less information can also be responsible for the poorer performance of the CNN solution, which is based on a learning process. To overcome the memory issues related to the 3D CNN solution, a 2.5 dimension approach could be used (Hering et al., 2019a). It has already been demonstrated to provide good registration results for 3D data and could help to improve the registration results by using larger input images. In this work, up to two sequences could be used to train the CNN solution, due to memory limitations. By using more powerful hardware, larger combinations of MRI protocols could include up to four sequences. It would be interesting to investigate whether the performance of the CNN method could improve. Moreover, a larger and more heterogeneous dataset could help to improve the performance of the deep learning method.

Another limitation is related to the landmarks provided in the BraTS-Reg dataset. Although this set provides more image pairs and landmarks than the Test Set, no information about the distance of the landmarks from the tumors is shared with the public. If the landmarks would be divided into two groups according to their distance from the pathology, it would be interesting to validate how the proposed methods perform in the different brain areas for this dataset.

## 5. Conclusions

To the best of our knowledge, our work provides the first quantitative analysis of the influence of different MRI sequences on the registration of longitudinal MRI data. We also evaluate how much impact the exclusion of the pathological tissues has on the registration of pre- and post-operative data. To conduct our experiments, a multi-level deep learning solution and an iterative method are proposed for the registration pre- and post-operative MRI data acquired in the neurosurgical context. A few changes have been made to the original CNN implementation (i) to accept multiparametric images and (ii) to mask specific tissues out of the distance measure. Our experiment showed that the best sequence to guide the registration process is the T1-CE. For the CNN solution, the combination of T1-CE and T2 sequences also leads to good results. The best performing solution in our experiments is provided by the iterative method, using the T1-CE sequence.

## Data availability statement

Publicly available datasets were analyzed in this study. This data can be found here: BraTS—https://sites.google.com/site/braintumorsegmentation/home/brats2015; BraTS-Reg—https://www.med.upenn.edu/cbica/brats-reg-challenge/.

## Author contributions

LC contributed to conception and design of the study. JK, BW, DW, FK, SS, and BM organized the databases and provided scientific support. JK, AH, SH, and HH provided scientific support regarding image registration and validation of the proposed algorithms. All authors contributed to manuscript revision, read, and approved the submitted version.

## Funding

This work was funded by the H2020 Marie-Curie ITN TRABIT (765148) project. LC is supported by the Translational Brain Imaging Training Network (TRABIT) under the European Union's ‘Horizon 2020' Research and Innovation Program (Grant agreement ID: 765148). SC is supported by National Institute Of Neurological Disorders and Stroke under project number R01NS112161. DW, JKi, and FK are supported by Deutsche Forschungsgemeinschaft (DFG) through TUM International Graduate School of Science and Engineering (IGSSE), GSC 81. JKi has received Grants from the ERC, DFG, BMBF and is Co-founder of Bonescreen GmbH. BM, BW, and FK are supported through the SFB 824, subproject B12.

## Conflict of interest

The authors declare that the research was conducted in the absence of any commercial or financial relationships that could be construed as a potential conflict of interest.

## Publisher's note

All claims expressed in this article are solely those of the authors and do not necessarily represent those of their affiliated organizations, or those of the publisher, the editors and the reviewers. Any product that may be evaluated in this article, or claim that may be made by its manufacturer, is not guaranteed or endorsed by the publisher.
